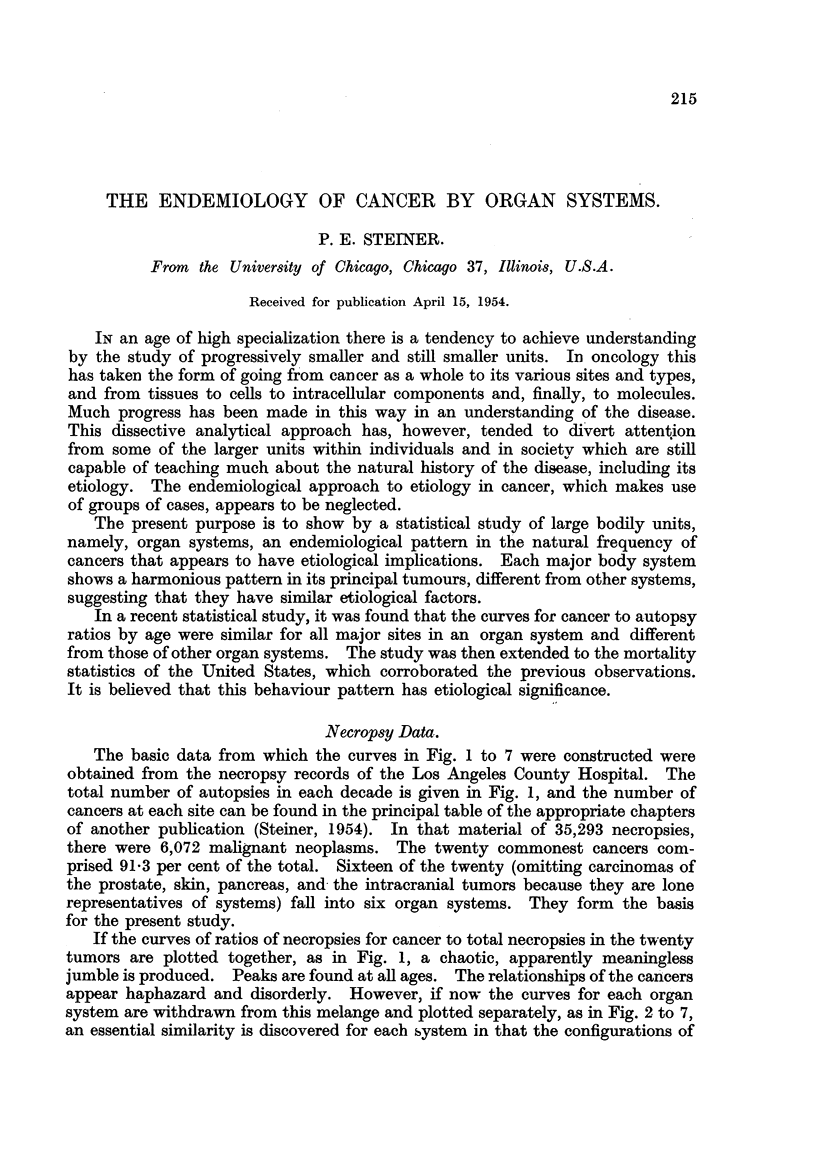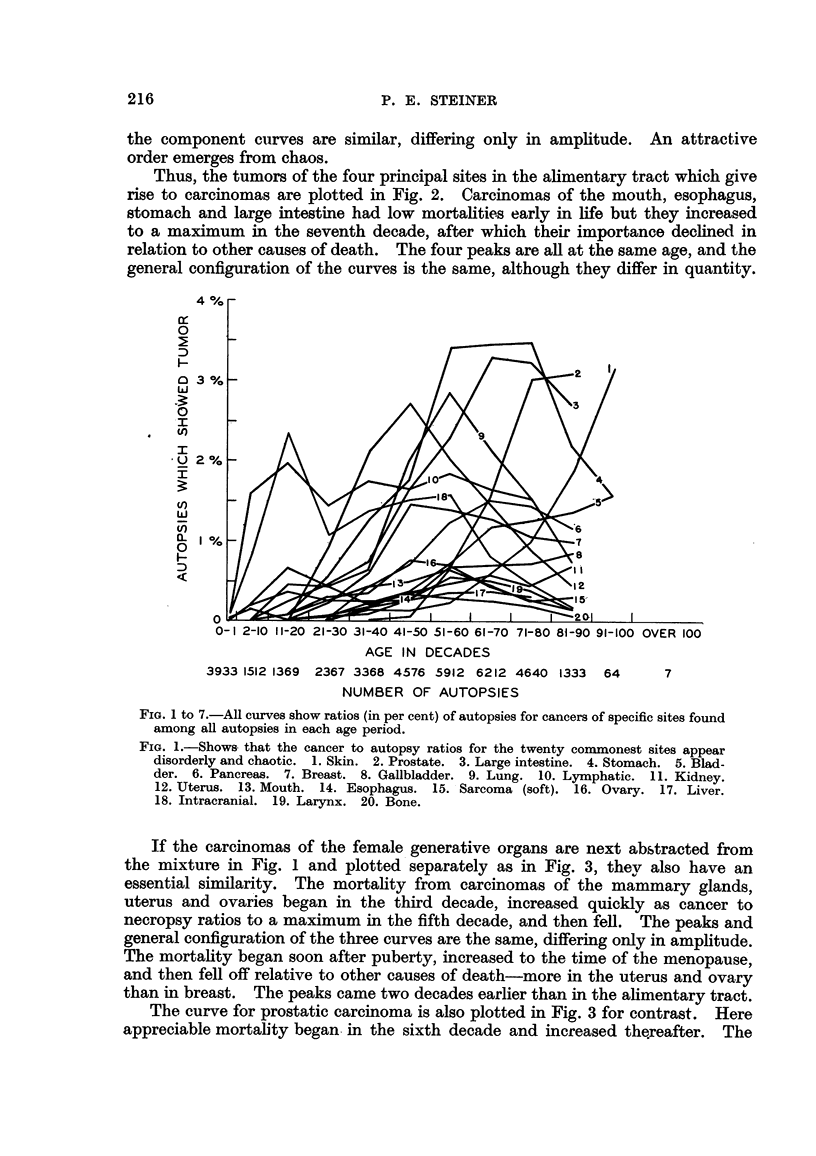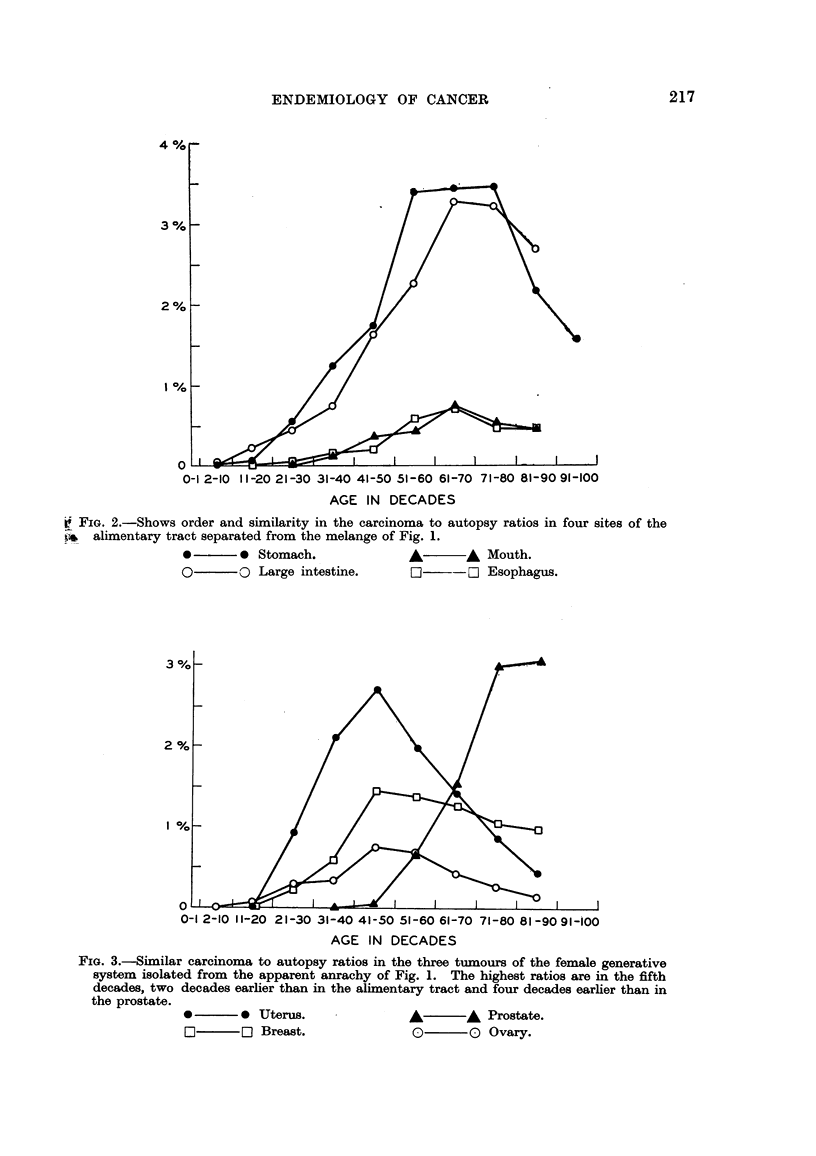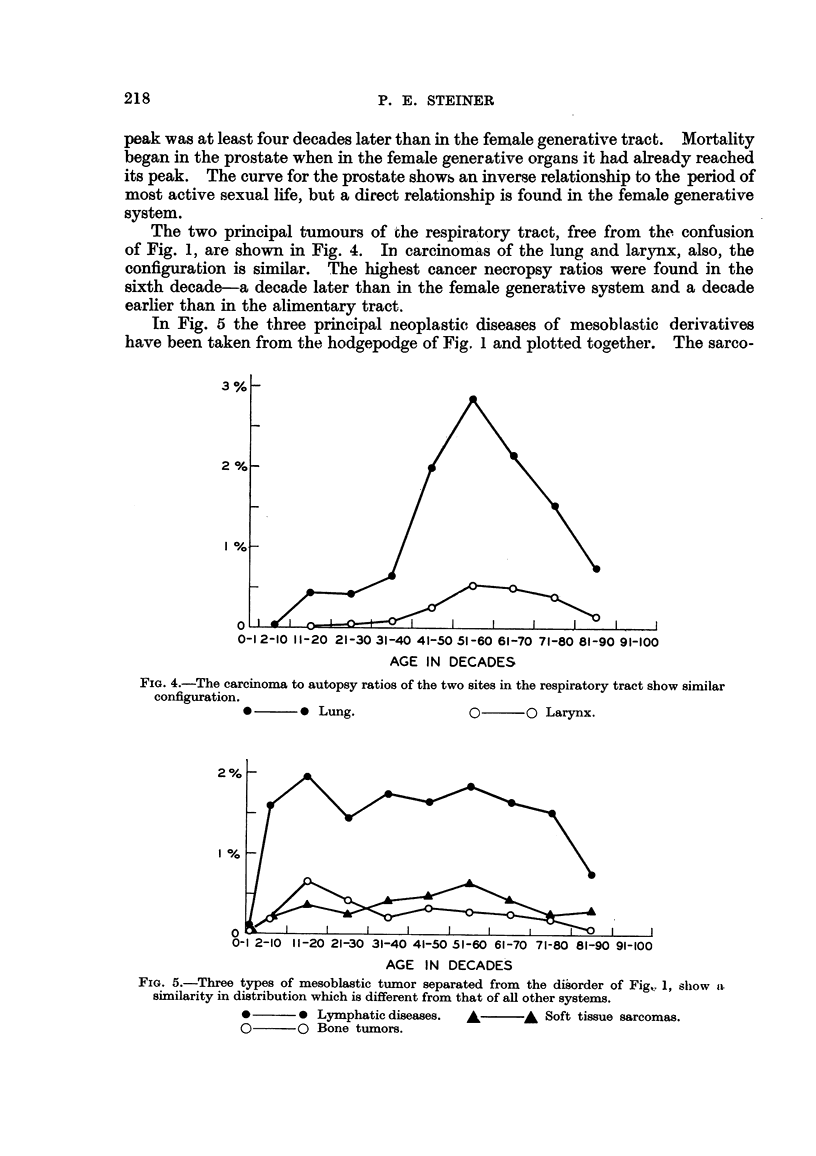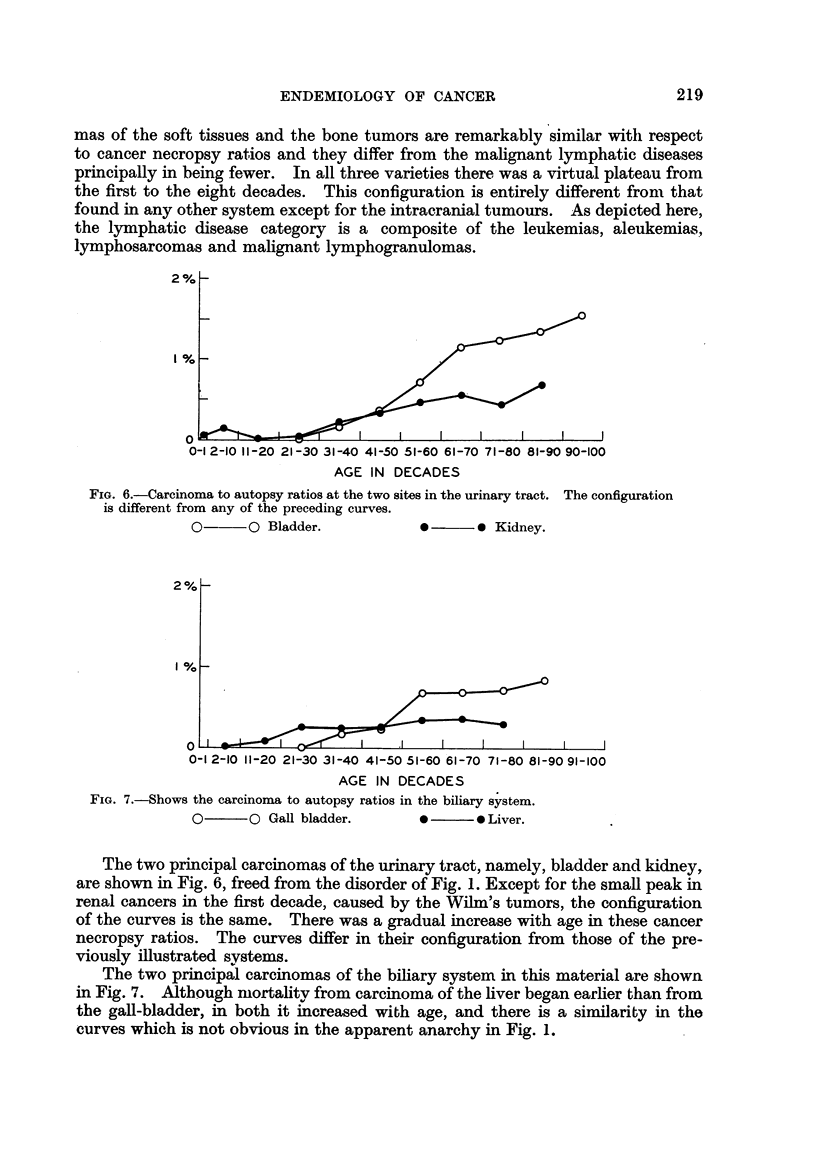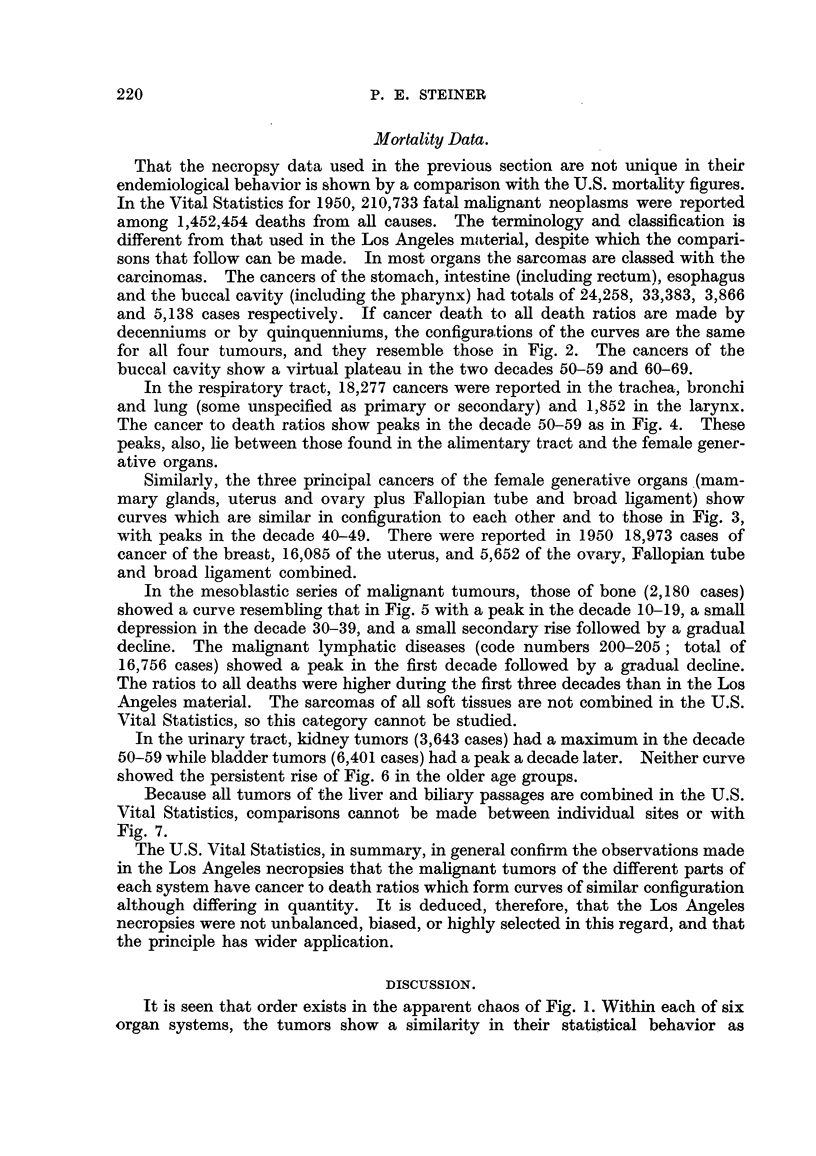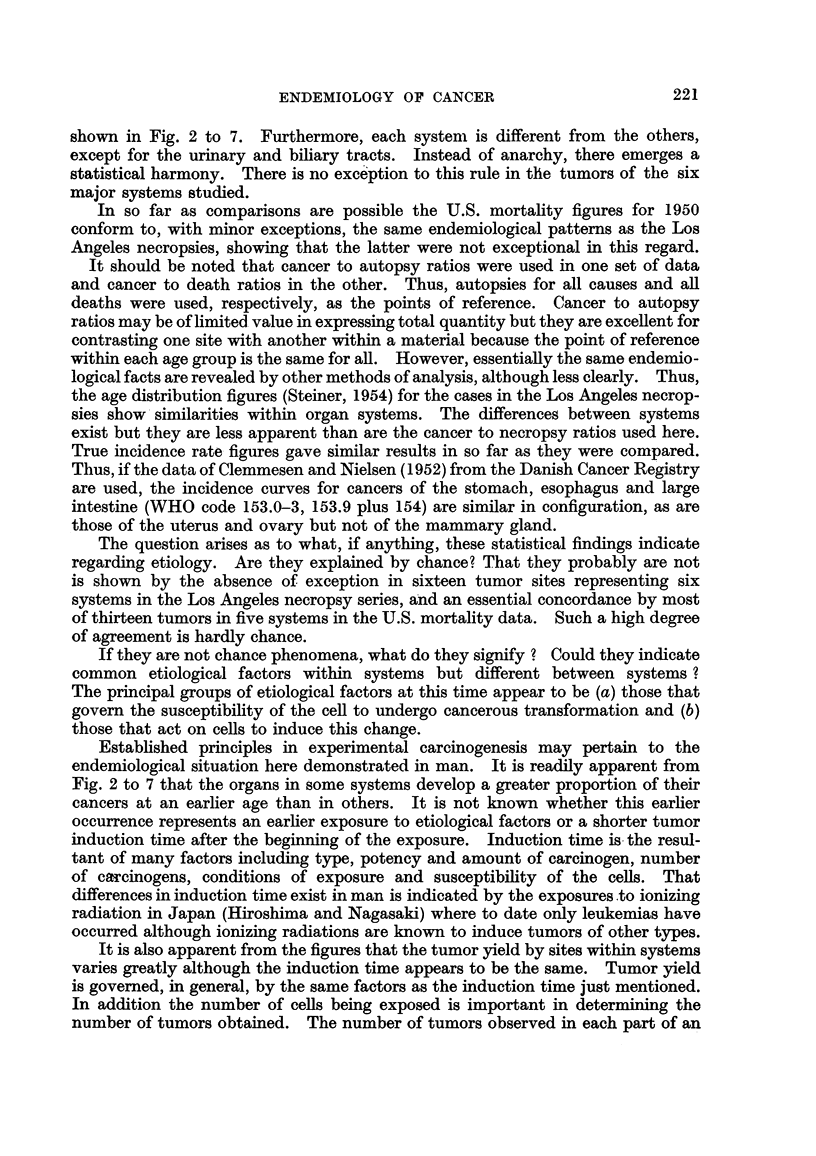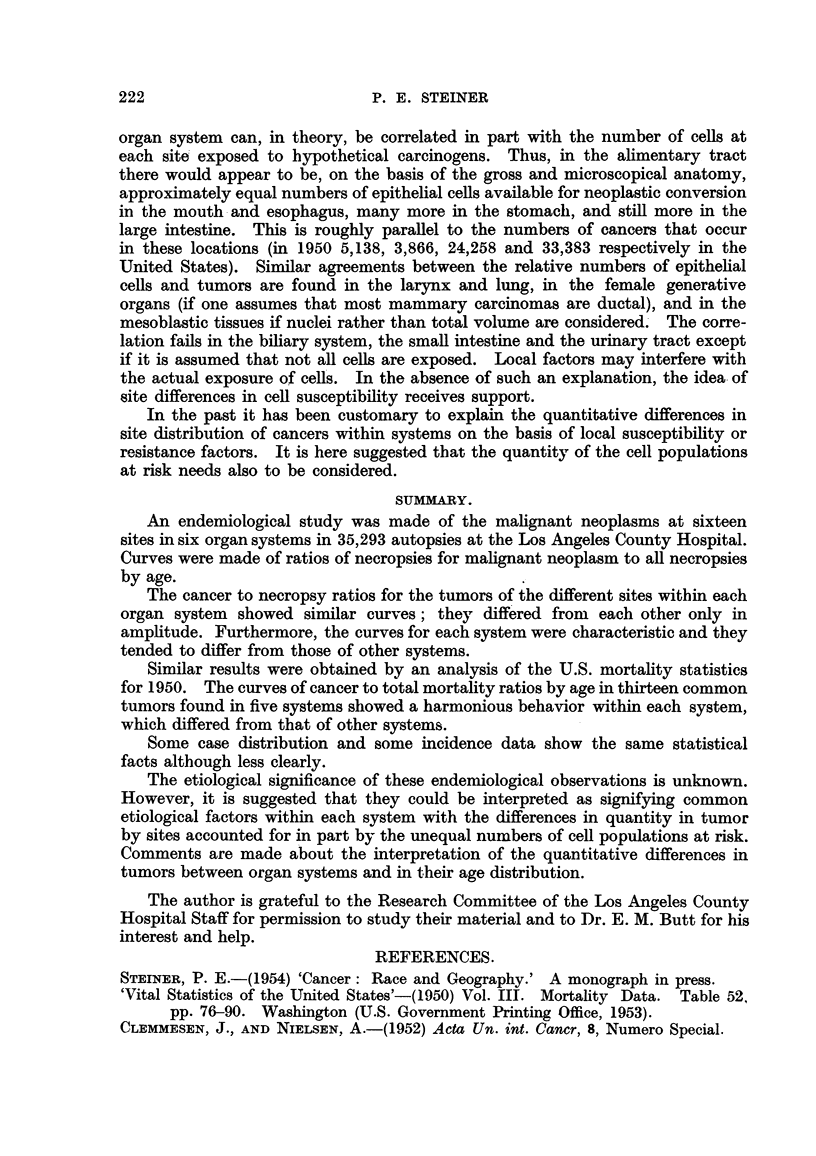# The Endemiology of Cancer of Organ Systems

**DOI:** 10.1038/bjc.1954.21

**Published:** 1954-06

**Authors:** P. E. Steiner


### THE ENDEMIOLOGY OF CANCER BY ORGAN SYSTEMS.

P. E. STEINER.

*From the University of Chicago, Chicago 37, Illinois, U.S.A.*

Received for publication April 15, 1954.

In an age of high specialization there is a tendency to achieve understanding by the study of progressively smaller and still smaller units. In oncology this has taken the form of going from cancer as a whole to its various sites and types, and from tissues to cells to intracellular components and, finally, to molecules. Much progress has been made in this way in an understanding of the disease. This dissective analytical approach has, however, tended to divert attention from some of the larger units within individuals and in society which are still capable of teaching much about the natural history of the disease, including its etiology. The endemiological approach to etiology in cancer, which makes use of groups of cases, appears to be neglected.

The present purpose is to show by a statistical study of large bodily units, namely, organ systems, an endemiological pattern in the natural frequency of cancers that appears to have etiological implications. Each major body system shows a harmonious pattern in its principal tumours, different from other systems, suggesting that they have similar etiological factors.

In a recent statistical study, it was found that the curves for cancer to autopsy ratios by age were similar for all major sites in an organ system and different from those of other organ systems. The study was then extended to the mortality statistics of the United States, which corroborated the previous observations. It is believed that this behaviour pattern has etiological significance.

### *Necropsy Data.*

The basic data from which the curves in Fig. 1 to 7 were constructed were obtained from the necropsy records of the Los Angeles County Hospital. The total number of autopsies in each decade is given in Fig. 1, and the number of cancers at each site can be found in the principal table of the appropriate chapters of another publication (Steiner, 1954). In that material of 35,293 necropsies, there were 6,072 malignant neoplasms. The twenty commonest cancers comprised 91·3 per cent of the total. Sixteen of the twenty (omitting carcinomas of the prostate, skin, pancreas, and the intracranial tumors because they are lone representatives of systems) fall into six organ systems. They form the basis for the present study.

If the curves of ratios of necropsies for cancer to total necropsies in the twenty tumors are plotted together, as in Fig. 1, a chaotic, apparently meaningless jumble is produced. Peaks are found at all ages. The relationships of the cancers appear haphazard and disorderly. However, if now the curves for each organ system are withdrawn from this melange and plotted separately, as in Fig. 2 to 7, an essential similarity is discovered for each system in that the configurations of the component curves are similar, differing only in amplitude. An attractive order emerges from chaos.

Thus, the tumors of the four principal sites in the alimentary tract which give rise to carcinomas are plotted in Fig. 2. Carcinomas of the mouth, esophagus, stomach and large intestine had low mortalities early in life but they increased to a maximum in the seventh decade, after which their importance declined in relation to other causes of death. The four peaks are all at the same age, and the general configuration of the curves is the same, although they differ in quantity.

FIG. 1 to 7.—All curves show ratios (in per cent) of autopsies for cancers of specific sites found among all autopsies in each age period.

FIG. 1.—Shows that the cancer to autopsy ratios for the twenty commonest sites appear disorderly and chaotic. 1. Skin. 2. Prostate. 3. Large intestine. 4. Stomach. 5. Bladder. 6. Pancreas. 7. Breast. 8. Gallbladder. 9. Lung. 10. Lymphatic. 11. Kidney. 12. Uterus. 13. Mouth. 14. Esophagus. 15. Sarcoma (soft). 16. Ovary. 17. Liver. 18. Intracranial. 19. Larynx. 20. Bone.

If the carcinomas of the female generative organs are next abstracted from the mixture in Fig. 1 and plotted separately as in Fig. 3, they also have an essential similarity. The mortality from carcinomas of the mammary glands, uterus and ovaries began in the third decade, increased quickly as cancer to necropsy ratios to a maximum in the fifth decade, and then fell. The peaks and general configuration of the three curves are the same, differing only in amplitude. The mortality began soon after puberty, increased to the time of the menopause, and then fell off relative to other causes of death—more in the uterus and ovary than in breast. The peaks came two decades earlier than in the alimentary tract.

The curve for prostatic carcinoma is also plotted in Fig. 3 for contrast. Here appreciable mortality began in the sixth decade and increased thereafter. The

FIG. 2.—Shows order and similarity in the carcinoma to autopsy ratios in four sites of the alimentary tract separated from the melange of Fig. 1.

●———● Stomach. ▲———▲ Mouth.

○———○ Large intestine. □———□ Esophagus.

FIG. 3.—Similar carcinoma to autopsy ratios in the three tumours of the female generative system isolated from the apparent anrachy of Fig. 1. The highest ratios are in the fifth decades, two decades earlier than in the alimentary tract and four decades earlier than in the prostate.

●———● Uterus. ▲———▲ Prostate.

□———□ Breast. ⊙———⊙ Ovary.

peak was at least four decades later than in the female generative tract. Mortality began in the prostate when in the female generative organs it had already reached its peak. The curve for the prostate shows an inverse relationship to the period of most active sexual life, but a direct relationship is found in the female generative system.

The two principal tumours of the respiratory tract, free from the confusion of Fig. 1, are shown in Fig. 4. In carcinomas of the lung and larynx, also, the configuration is similar. The highest cancer necropsy ratios were found in the sixth decade—a decade later than in the female generative system and a decade earlier than in the alimentary tract.

In Fig. 5 the three principal neoplastic diseases of mesoblastic derivatives have been taken from the hodgepodge of Fig. 1 and plotted together. The sarco-

FIG. 4.—The carcinoma to autopsy ratios of the two sites in the respiratory tract show similar configuration.

● ——— ● Lung. ○ ——— ○ Larynx.

FIG. 5.—Three types of mesoblastic tumor separated from the disorder of Fig. 1, show a similarity in distribution which is different from that of all other systems.

● ——— ● Lymphatic diseases. ▲ ——— ▲ Soft tissue sarcomas.

○ ——— ○ Bone tumors.

mas of the soft tissues and the bone tumors are remarkably similar with respect to cancer necropsy ratios and they differ from the malignant lymphatic diseases principally in being fewer. In all three varieties there was a virtual plateau from the first to the eight decades. This configuration is entirely different from that found in any other system except for the intracranial tumours. As depicted here, the lymphatic disease category is a composite of the leukemias, aleukemias, lymphosarcomas and malignant lymphogranulomas.

FIG. 6.—Carcinoma to autopsy ratios at the two sites in the urinary tract. The configuration is different from any of the preceding curves.

O———O Bladder. ●———● Kidney.

FIG. 7.—Shows the carcinoma to autopsy ratios in the biliary system.

O———O Gall bladder. ●———● Liver.

The two principal carcinomas of the urinary tract, namely, bladder and kidney, are shown in Fig. 6, freed from the disorder of Fig. 1. Except for the small peak in renal cancers in the first decade, caused by the Wilm's tumors, the configuration of the curves is the same. There was a gradual increase with age in these cancer necropsy ratios. The curves differ in their configuration from those of the previously illustrated systems.

The two principal carcinomas of the biliary system in this material are shown in Fig. 7. Although mortality from carcinoma of the liver began earlier than from the gall-bladder, in both it increased with age, and there is a similarity in the curves which is not obvious in the apparent anarchy in Fig. 1.

*Mortality Data.*

That the necropsy data used in the previous section are not unique in their endemiological behavior is shown by a comparison with the U.S. mortality figures. In the Vital Statistics for 1950, 210,733 fatal malignant neoplasms were reported among 1,452,454 deaths from all causes. The terminology and classification is different from that used in the Los Angeles material, despite which the comparisons that follow can be made. In most organs the sarcomas are classed with the carcinomas. The cancers of the stomach, intestine (including rectum), esophagus and the buccal cavity (including the pharynx) had totals of 24,258, 33,383, 3,866 and 5,138 cases respectively. If cancer death to all death ratios are made by decenniums or by quinquenniums, the configurations of the curves are the same for all four tumours, and they resemble those in Fig. 2. The cancers of the buccal cavity show a virtual plateau in the two decades 50–59 and 60–69.

In the respiratory tract, 18,277 cancers were reported in the trachea, bronchi and lung (some unspecified as primary or secondary) and 1,852 in the larynx. The cancer to death ratios show peaks in the decade 50–59 as in Fig. 4. These peaks, also, lie between those found in the alimentary tract and the female generative organs.

Similarly, the three principal cancers of the female generative organs (mammary glands, uterus and ovary plus Fallopian tube and broad ligament) show curves which are similar in configuration to each other and to those in Fig. 3, with peaks in the decade 40–49. There were reported in 1950 18,973 cases of cancer of the breast, 16,085 of the uterus, and 5,652 of the ovary, Fallopian tube and broad ligament combined.

In the mesoblastic series of malignant tumours, those of bone (2,180 cases) showed a curve resembling that in Fig. 5 with a peak in the decade 10–19, a small depression in the decade 30–39, and a small secondary rise followed by a gradual decline. The malignant lymphatic diseases (code numbers 200–205 ; total of 16,756 cases) showed a peak in the first decade followed by a gradual decline. The ratios to all deaths were higher during the first three decades than in the Los Angeles material. The sarcomas of all soft tissues are not combined in the U.S. Vital Statistics, so this category cannot be studied.

In the urinary tract, kidney tumors (3,643 cases) had a maximum in the decade 50–59 while bladder tumors (6,401 cases) had a peak a decade later. Neither curve showed the persistent rise of Fig. 6 in the older age groups.

Because all tumors of the liver and biliary passages are combined in the U.S. Vital Statistics, comparisons cannot be made between individual sites or with Fig. 7.

The U.S. Vital Statistics, in summary, in general confirm the observations made in the Los Angeles necropsies that the malignant tumors of the different parts of each system have cancer to death ratios which form curves of similar configuration although differing in quantity. It is deduced, therefore, that the Los Angeles necropsies were not unbalanced, biased, or highly selected in this regard, and that the principle has wider application.

### DISCUSSION.

It is seen that order exists in the apparent chaos of Fig. 1. Within each of six organ systems, the tumors show a similarity in their statistical behavior as shown in Fig. 2 to 7. Furthermore, each system is different from the others, except for the urinary and biliary tracts. Instead of anarchy, there emerges a statistical harmony. There is no exception to this rule in the tumors of the six major systems studied.

In so far as comparisons are possible the U.S. mortality figures for 1950 conform to, with minor exceptions, the same endemiological patterns as the Los Angeles necropsies, showing that the latter were not exceptional in this regard.

It should be noted that cancer to autopsy ratios were used in one set of data and cancer to death ratios in the other. Thus, autopsies for all causes and all deaths were used, respectively, as the points of reference. Cancer to autopsy ratios may be of limited value in expressing total quantity but they are excellent for contrasting one site with another within a material because the point of reference within each age group is the same for all. However, essentially the same endemiological facts are revealed by other methods of analysis, although less clearly. Thus, the age distribution figures (Steiner, 1954) for the cases in the Los Angeles necropsies show similarities within organ systems. The differences between systems exist but they are less apparent than are the cancer to necropsy ratios used here. True incidence rate figures gave similar results in so far as they were compared. Thus, if the data of Clemmesen and Nielsen (1952) from the Danish Cancer Registry are used, the incidence curves for cancers of the stomach, esophagus and large intestine (WHO code 153.0–3, 153.9 plus 154) are similar in configuration, as are those of the uterus and ovary but not of the mammary gland.

The question arises as to what, if anything, these statistical findings indicate regarding etiology. Are they explained by chance? That they probably are not is shown by the absence of exception in sixteen tumor sites representing six systems in the Los Angeles necropsy series, and an essential concordance by most of thirteen tumors in five systems in the U.S. mortality data. Such a high degree of agreement is hardly chance.

If they are not chance phenomena, what do they signify ? Could they indicate common etiological factors within systems but different between systems ? The principal groups of etiological factors at this time appear to be (*a*) those that govern the susceptibility of the cell to undergo cancerous transformation and (*b*) those that act on cells to induce this change.

Established principles in experimental carcinogenesis may pertain to the endemiological situation here demonstrated in man. It is readily apparent from Fig. 2 to 7 that the organs in some systems develop a greater proportion of their cancers at an earlier age than in others. It is not known whether this earlier occurrence represents an earlier exposure to etiological factors or a shorter tumor induction time after the beginning of the exposure. Induction time is the resultant of many factors including type, potency and amount of carcinogen, number of carcinogens, conditions of exposure and susceptibility of the cells. That differences in induction time exist in man is indicated by the exposures to ionizing radiation in Japan (Hiroshima and Nagasaki) where to date only leukemias have occurred although ionizing radiations are known to induce tumors of other types.

It is also apparent from the figures that the tumor yield by sites within systems varies greatly although the induction time appears to be the same. Tumor yield is governed, in general, by the same factors as the induction time just mentioned. In addition the number of cells being exposed is important in determining the number of tumors obtained. The number of tumors observed in each part of an organ system can, in theory, be correlated in part with the number of cells at each site exposed to hypothetical carcinogens. Thus, in the alimentary tract there would appear to be, on the basis of the gross and microscopical anatomy, approximately equal numbers of epithelial cells available for neoplastic conversion in the mouth and esophagus, many more in the stomach, and still more in the large intestine. This is roughly parallel to the numbers of cancers that occur in these locations (in 1950 5,138, 3,866, 24,258 and 33,383 respectively in the United States). Similar agreements between the relative numbers of epithelial cells and tumors are found in the larynx and lung, in the female generative organs (if one assumes that most mammary carcinomas are ductal), and in the mesoblastic tissues if nuclei rather than total volume are considered. The correlation fails in the biliary system, the small intestine and the urinary tract except if it is assumed that not all cells are exposed. Local factors may interfere with the actual exposure of cells. In the absence of such an explanation, the idea of site differences in cell susceptibility receives support.

In the past it has been customary to explain the quantitative differences in site distribution of cancers within systems on the basis of local susceptibility or resistance factors. It is here suggested that the quantity of the cell populations at risk needs also to be considered.

### SUMMARY.

An endemiological study was made of the malignant neoplasms at sixteen sites in six organ systems in 35,293 autopsies at the Los Angeles County Hospital. Curves were made of ratios of necropsies for malignant neoplasm to all necropsies by age.

The cancer to necropsy ratios for the tumors of the different sites within each organ system showed similar curves; they differed from each other only in amplitude. Furthermore, the curves for each system were characteristic and they tended to differ from those of other systems.

Similar results were obtained by an analysis of the U.S. mortality statistics for 1950. The curves of cancer to total mortality ratios by age in thirteen common tumors found in five systems showed a harmonious behavior within each system, which differed from that of other systems.

Some case distribution and some incidence data show the same statistical facts although less clearly.

The etiological significance of these endemiological observations is unknown. However, it is suggested that they could be interpreted as signifying common etiological factors within each system with the differences in quantity in tumor by sites accounted for in part by the unequal numbers of cell populations at risk. Comments are made about the interpretation of the quantitative differences in tumors between organ systems and in their age distribution.

The author is grateful to the Research Committee of the Los Angeles County Hospital Staff for permission to study their material and to Dr. E. M. Butt for his interest and help.

### REFERENCES.

Steiner, P. E.—(1954) 'Cancer: Race and Geography.' A monograph in press.

'Vital Statistics of the United States'—(1950) Vol. III. Mortality Data. Table 52, pp. 76–90. Washington (U.S. Government Printing Office, 1953).

Clemmesen, J., and Nielsen, A.—(1952) *Acta Un. int. Cancr*, **8**, Numero Special.